# Microsurgical resection of an anterior medullary arteriovenous malformation

**DOI:** 10.3171/2020.10.FOCVID2073

**Published:** 2021-01-01

**Authors:** Joshua S. Catapano, Rohin Singh, Visish M. Srinivasan, Michael T. Lawton

**Affiliations:** Department of Neurosurgery, Barrow Neurological Institute, St. Joseph’s Hospital and Medical Center, Phoenix, Arizona

**Keywords:** anterior medullary AVM, arteriovenous malformation

## Abstract

Arteriovenous malformations (AVMs) in the brainstem, specifically medullary AVMs, are exceedingly rare and difficult to treat. These lesions are commonly more aggressive than supratentorial AVMs and pose their own unique treatment challenges. Current treatment options for these AVMs consist of endovascular embolization or open surgery. Radiosurgery is not favored because it is associated with potential risk to the brainstem and lower obliteration rates. Here the authors report the case of a 27-year-old man with a ruptured anterior medullary AVM. The patient underwent a successful far-lateral craniotomy for resection of the AVM.

The video can be found here: https://youtu.be/lyOfOQ3sBdU

## Transcript

**0:20** This video will demonstrate the microsurgical resection of an anterior medullary arteriovenous malformation.^1–5^

**0:28** The patient is a 27-year-old man. He presented with a sudden severe headache and right hemiplegia. He was responsive on exam and able to follow commands only on the left side.

**0:41** Here are his imaging findings. On the left, you see a CT angiogram with a hematoma in the substance of the medulla. In the middle, you see a left vertebral artery angiogram, which demonstrates a feeding artery from the left V_4_ segment of the vertebral artery. On the right, you can see a right vertebral artery angiogram with filling of the anterior spinal artery, which descends down to supply the AVM from above.

**1:12** His Spetzler-Martin grade was III, with 1 point each for size, deep venous drainage, and eloquence. His Lawton-Young supplementary grade was II, with all the points given for his age. His supplemented Spetzler-Martin grade was 5.

**1:27** Here is an image showing the three-quarter prone position, the hockey stick skin incision, and the far-lateral craniotomy with C1 laminectomy. This gives excellent exposure of the left V_4_ segment of the vertebral artery and access to the anterior medulla.

**1:46** Key surgical steps include extensive condylectomy with dissection of the vertebral artery from the sulcus arteriosus to the dural ring. This was needed to gain access to the anterior medulla. Dentate ligaments were cut, also giving good access to the V_4_ segment. A large feeder from the left V_4_ vertebral artery was divided. A large feeder from the right anterior spinal artery was divided. The AVM was circumferentially dissected and removed, and IC green video angiography confirmed a complete resection.

**2:18** Here is an overview of the condylectomy. You can see the dissection all the way deep into the condyle, and you can also see removal of bone around the vertebral artery in its sulcus arteriosus. There was significant soft tissue around the artery, and this was extensively dissected so that the exposure would be flush against the lateral portion of the clivus. Additional bone was removed on the contralateral side of the foramen magnum to widen this exposure. Here is the midline dural incision and the flap raised against the condyle. You can see how flat that dural flap is against the condyle. This allows access to the dentate ligaments, which are cut here.

**3:03** You can see the C1 nerve rootlet here is cauterized and divided as it joins the vertebral artery at its dural ring. Now we have excellent access along the vertebral artery and medial to it along the clivus. You can see this large branch from the V_4_ segment as it comes off of the vertebral artery and joins the nidus. Here are a couple of smaller branches, and these are coagulated and separated from their supply along that V_4_ segment.

**3:39** This was one of two major feeders to the AVM nidus. Now that the dissection turns more medially, we are going to the anterior surface of the medulla. You can see a dilated draining vein there on the surface. And now we have feeding arteries from the anterior spinal artery that are coming into view.

**3:57** This was an embolized vessel here that was divided. These are some of the branches from the V_4_ segment that are being cauterized. And this cauterization just helps to shrink the nidus down on its pial surface, on the medulla. As we work further forward, we bring the anterior spinal artery into view.

**4:22** The anterior spinal artery was what was supplying the nidus from the front. You can see there is still some significant shunting into the nidus even after dividing the anterior spinal artery. So, some additional dissection along the margins of the nidus was needed to de-arterialize it.

**4:47** Only those branches that could be seen joining the AVM on the pial surface were cauterized and divided, as shown here. You can see the vein on the front of the medulla, which is starting to darken in color. That bluish color is an indication that we are finally getting to the point where the AVM has been de-arterialized. So, an additional run shows that the contributions from the V_4_ segment are quieted. Contributions from the anterior spinal artery are also quieted, so that now we can take this draining vein and cauterize it.

**5:31** This allows us to go around this medial border of the AVM and to free it up completely and remove it. You can see how this is a nice example of an AVM on the anterior surface of the medulla that is on the pia only and does not invade into the parenchyma of the medulla.

**5:54** The patient was neurologically unchanged after surgery, with his preexisting hemiplegia. He remained in the intensive care unit for just over a week and was transferred to long-term acute care on postoperative day 9.

**6:09** Here are his postoperative images, which show the extent of the condylectomy on the CT scan on the left. His vertebral artery angiograms in the center and right show that the AVM no longer fills. Those feeding arteries from the left V_4_ segment and also from the right anterior spinal artery have been nicely occluded. You can see preservation of the anterior spinal artery on that right side of the image perfusing down the cord.

**6:37** In conclusion, these anterior medullary AVMs are exceedingly rare. I have only seen a handful in my experience. These lesions tend to be more devastating with hemorrhage than those located in the supratentorial space. These anterior medullar AVMs require an extensive far-lateral craniotomy to access these anteriorly located feeders. AVM resection is preferred, but occlusion in situ may be a safer alternative for complex AVMs, where it is difficult to see all of the features. Though associated with risk, surgical resection remains the treatment option with the highest and most immediate cure rate. Thank you.

## Acknowledgments

The authors thank the staff of Neuroscience Publications at Barrow Neurological Institute for assistance with transcript and video preparation.

## Author Contributions

Primary surgeon: Lawton. Editing and drafting the video and abstract: all authors. Critically revising the work: Lawton, Srinivasan. Reviewed submitted version of the work: Lawton, Srinivasan.

## References

[1] Han SJ Englot DJ Kim H Lawton MT Brainstem arteriovenous malformations: anatomical subtypes, assessment of “occlusion in situ” technique, and microsurgical results J Neurosurg 2015 122 1 107 117 25343188 10.3171/2014.8.JNS1483 PMC5261863

[2] Madhugiri VS Teo MKC Vavao J Brainstem arteriovenous malformations: lesion characteristics and treatment outcomes J Neurosurg 2018 128 1 126 136 28298018 10.3171/2016.9.JNS16943

[3] Ito M Yamamoto T Mishina H Arteriovenous malformation of the medulla oblongata supplied by the anterior spinal artery in a child: treatment by microsurgical obliteration of the feeding artery Pediatr Neurosurg 2000 33 6 293 297 11182639 10.1159/000055974

[4] Lawton MT Seven AVMs: Tenets and Techniques for Resection Thieme 2014

[5] Yang W Porras JL Garzon-Muvdi T Management outcome of brainstem arteriovenous malformations: the role of radiosurgery World Neurosurg 2016 94 64 72 27368509 10.1016/j.wneu.2016.06.082

